# Matcha green tea (MGT) inhibits the propagation of cancer stem cells (CSCs), by targeting mitochondrial metabolism, glycolysis and multiple cell signalling pathways

**DOI:** 10.18632/aging.101483

**Published:** 2018-08-23

**Authors:** Gloria Bonuccelli, Federica Sotgia, Michael P. Lisanti

**Affiliations:** 1 Translational Medicine, School of Environment and Life Sciences, Biomedical Research Centre, University of Salford, Greater Manchester, United Kingdom

**Keywords:** Matcha green tea, cancer stem-like cells (CSCs), proteomics analysis, metabolism, mitochondrial OXPHOS, glycolysis

## Abstract

Matcha green tea (MGT) is a natural product that is currently used as a dietary supplement and may have significant anti-cancer properties. However, the molecular mechanism(s) underpinning its potential health benefits remain largely unknown. Here, we used MCF7 cells (an ER(+) human breast cancer cell line) as a model system, to systematically dissect the effects of MGT at the cellular level, via i) metabolic phenotyping and ii) unbiased proteomics analysis. Our results indicate that MGT is indeed sufficient to inhibit the propagation of breast cancer stem cells (CSCs), with an IC-50 of ~0.2 mg/ml, in tissue culture. Interestingly, metabolic phenotyping revealed that treatment with MGT is sufficient to suppress both oxidative mitochondrial metabolism (OXPHOS) and glycolytic flux, shifting cancer cells towards a more quiescent metabolic state. Unbiased label-free proteomics analysis identified the specific mitochondrial proteins and glycolytic enzymes that were down-regulated by MGT treatment. Moreover, to discover the underlying signalling pathways involved in this metabolic shift, we subjected our proteomics data sets to bio-informatics interrogation via Ingenuity Pathway Analysis (IPA) software. Our results indicate that MGT strongly affected mTOR signalling, specifically down-regulating many components of the 40S ribosome. This raises the intriguing possibility that MGT can be used as inhibitor of mTOR, instead of chemical compounds, such as rapamycin. In addition, other key pathways were affected, including the anti-oxidant response, cell cycle regulation, as well as interleukin signalling. Our results are consistent with the idea that MGT may have significant therapeutic potential, by mediating the metabolic reprogramming of cancer cells.

## Introduction

Breast cancer is a leading cause of women’s cancers, although a small fraction of men may also be affected [1]. In this context, tumor recurrence [2] is particularly lethal and in the advanced stages is associated with treatment failure, due to therapy resistance [3,4]. As current treatment strategies often tend to be associated with serious side effects, new non-toxic treatment strategies would fulfil an unmet medical need. As a consequence, many translational scientists and clinicians are actively involved in the search for novel natural products, including spices and herbs, that may have therapeutic value and confer patient benefit.

Interestingly, several recent studies have suggested that Japanese green tea has anti-proliferative, anti-oxidant, anti-bacterial, and chemo-preventive effects [5,6]. In direct support of this notion, it has been demonstrated that a key component of green tea, namely epigallocatechin-3-gallate, behaves as an anti-oxidant and shows anti-tumor effects against breast cancer cells [7,8]. Green tea may also interact with other components of the diet, including those natural products contained within soy and mushrooms [9,10]. Consumption of green tea could partially explain why Asian-American women show a decreased risk for developing breast cancer [11].

Recently, the therapeutic effects of green tea catechins on breast tumorigenesis were evaluated out using pre-clinical models, as well as clinical trials [12-15]. Furthermore, a meta-analysis of epidemiological studies in 2006 suggested that green tea may lower the risk of developing colorectal cancer [16]. Recently, it was demonstrated that tea has other protective effects, such as reducing the risk of mortality from heart disease and stroke (cerebrovascular accident) [17].

A subsequent study, using green tea supplements, supports the idea that green tea extracts could be used in the chemo-prevention of metachronous colorectal adenomas, in Korean patients [18] (ClinicalTrials.gov, number NCT02321969). Finally, green tea has shown positive benefits in reducing Acute Myeloid Leukaemia (AML) risk [19] and prostate cancer [20]. Taken together, these studies suggest that green tea or its components, may be very useful as anti-cancer agents.

Several published preliminary studies in breast cancer have also been conducted, to evaluate the mechanism of action of green tea components. A broad-spectrum of different mechanism(s) has been described. For example, these data suggest that green tea components display the following regulatory properties: i) anti-angiogenic effects, mediated through the inhibition of HIF1-α and NF-kB [21]; and ii) anti-inflammatory effects, mediated by inhibiting the infiltration of Tumor-Associated Macrophages and the secretion of IL-6 and TNF-α [22]; iii) as well as the up-regulation of apoptosis and the down-regulation of cell proliferation [23], among others.

Here, we investigated the potential therapeutic effects of Matcha green tea (MGT), a natural product, on the propagation of breast cancer stem cells, by using MCF7 cells as a model system. Our results indicate that MGT effectively inhibits the propagation of CSCs, as measured using the mammosphere assay, with an IC-50 of 0.2 mg/ml. Importantly, previous studies have clearly demonstrated that CSCs are involved in mediating tumor recurrence, distant metastasis and therapy failure due to chemo- and radio-resistance [24-26].

In this report, we focused on following the metabolic effects of MGT on breast cancer cells, by using the Seahorse XFe96 Analyzer, to quantitatively measure metabolic flux. Our results directly show that MGT reduces both mitochondrial metabolism, as well as glycolysis, maintaining cancer cells in a more metabolically quiescent state. Unbiased proteomics analysis identified the mitochondrial proteins and glycolytic enzymes that were down-regulated by MGT treatment. Thus, MGT or its components, may possess significant anti-cancer activity, by mediating the metabolic reprogramming of cancer cells.

## RESULTS

**Figure 1:**
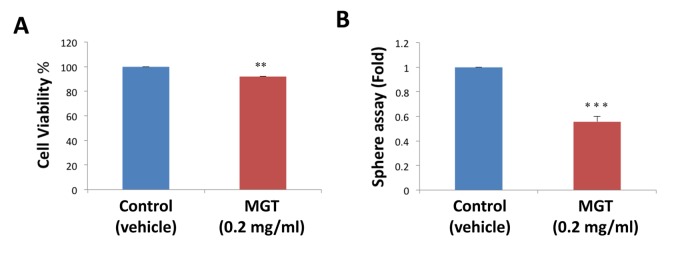
**MGT treatment reduces stemness in MCF7 breast cancer cells.** (**A**) The effects of 0.2 mg/ml MGT on cell proliferation were tested on MCF7 cells in monolayer by SRB assay. Note that MGT only slightly reduced viability of bulk cancer cells by 8%. (**B**) Importantly, MGT inhibited the sphere-forming ability of MCF7 cells by 50%. Bar graphs are shown as the mean ± SEM; *t*-test, two-tailed test. **p < 0.005, ***p < 0.0001.

Here, to dissect the effects of MGT at the phenotypic cellular level, MCF7 cancer cells were treated in adherent conditions with 0.2 mg/ml MGT, starting 24 hours after plating. MGT treatment showed only a slight effect on the cancer cells in monolayer, reducing viability of bulk cancer cells by 8% (Fig. 1A). Next, to assess the stem cell activity and self-renewal, MCF7 cells were cultured in low-attachment conditions in the presence or absence of MGT. After five days of culture, the ability of CSCs to survive and grow was evaluated by counting the number of mammospheres. Intriguingly, MGT treatment inhibited the sphere-formation capacity of MCF7 cells by 50%, indicating that MGT halts the propagation of cancer stem cells (Fig. 1B).

**Figure 2:**
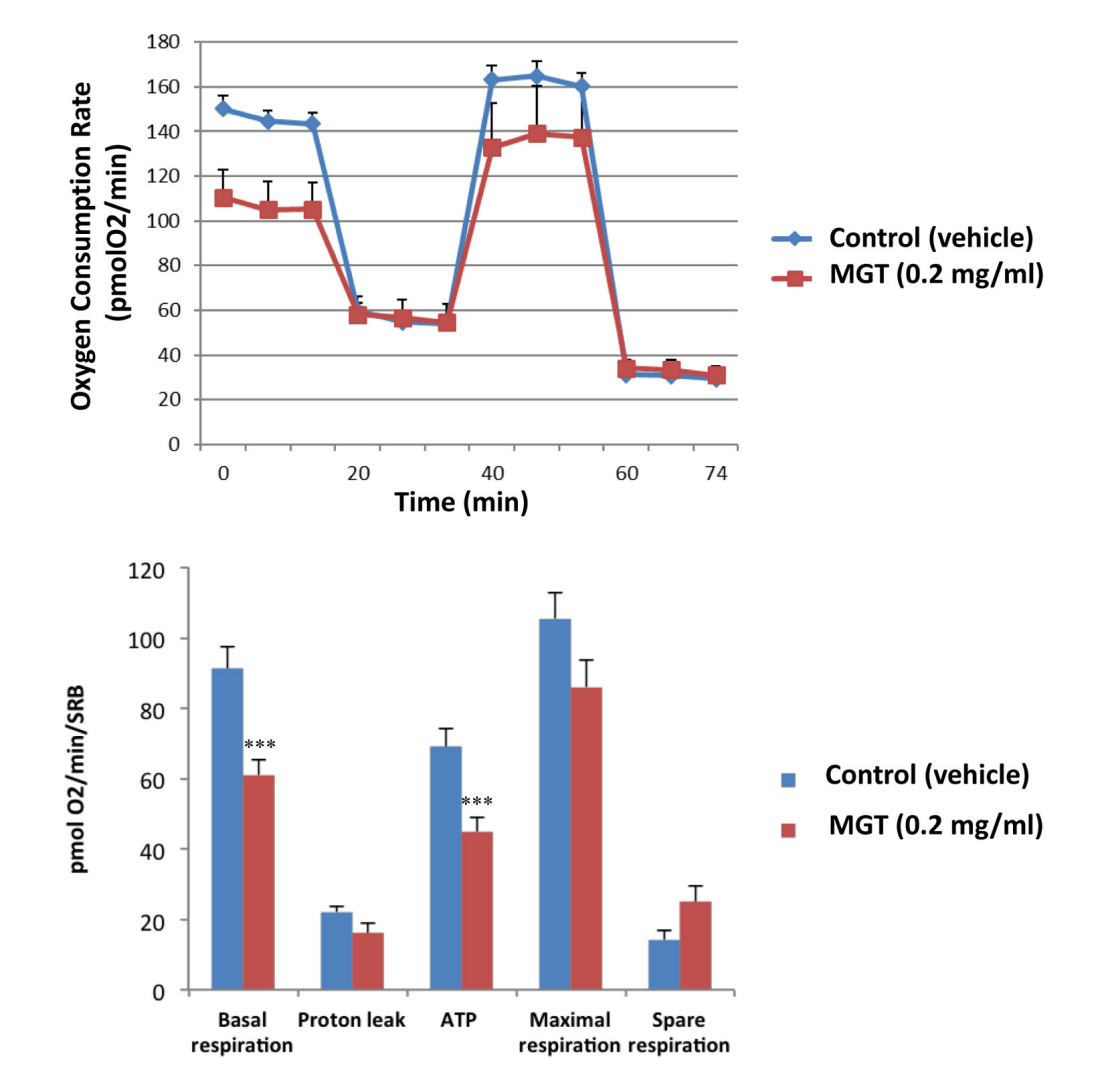
**MGT treatment reduces basal respiration and ATP production in MCF7 cells.** MCF7 cells were seeded at the density of six thousands cells in 96-wells plates. After twenty-four hours, filtered 0.2 mg/ml MGT was added and incubated for seventy-two hours. Oxygen consumption rate (OCR) was measured by Seahorse XF Analyser. Top panel: representative trace; bottom panel: bar graph with OCR quantification. Note that MGT treatment significantly decreases the basal respiration and the ATP production as compared to control cells. Others parameters, such as proton leak, maximal and spare respirations did not significantly change. Experiments were performed 3 times, with six repeats for each replicate. Bar graphs are shown as the mean ± SEM; *t*-test, two-tailed test. ***p < 0.0001.

**Figure 3:**
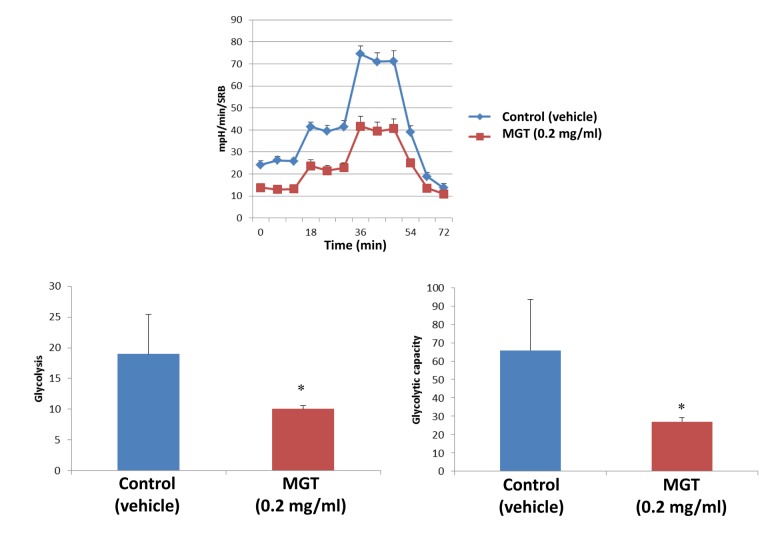
**MGT treatment inhibits glycolysis of MCF7 cells.** MCF7 cells were seeded and treated with MGT as described above. Extracellular consumption rate (ECAR) was assessed by Seahorse XF Analyser. Top panel: representative trace; bottom panel: bar graph with OCR quantification. Importantly, the treatment significantly reduced the glycolysis and the glycolytic capacity as compared to control cells. Experiments were performed in triplicate, six repeats for each replicate. Bar graphs are shown as the mean ± SEM, *t*-test, two-tailed test. *p < 0.05.

It is well known that the propagation of CSCs depends, at least partially, on increased mitochondrial biogenesis and metabolism [27,28]. To evaluate a possible effect of MGT on cellular metabolic features, we employed the Seahorse XF Analyzer to measure oxygen consumption rate (OCR) and extracellular acidification rate (ECAR). Interestingly, a 72-hours treatment with MGT significantly decreased basal respiration and ATP production in MCF7 breast cancer cells, as compared to untreated cells (Fig. 2). Moreover, MGT treated cells displayed a reduction of glycolytic rate and glycolytic capacity, the latter referred to as measurement of the maximal capacity of the cells to respond to a higher ATP demand (Fig. 3).

**Figure 4:**
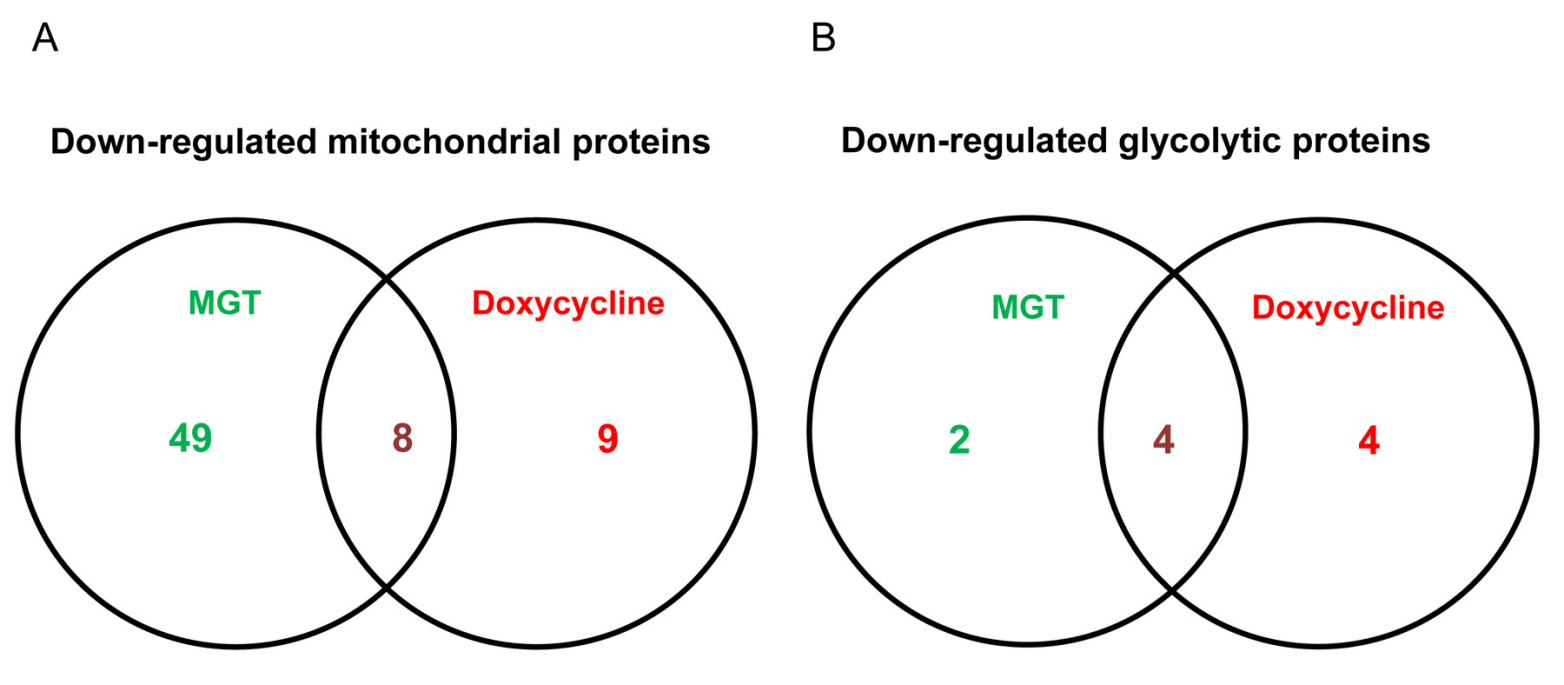
**Venn diagram of proteomics data of MGT-treated MCF7 cells *versus* doxycycline-treated MCF7 cells.** Proteomic analysis validates the metabolic effects of MGT on breast cancer cells. (A) Venn diagram of mitochondrial down-regulated proteins in MGT-treated cells versus doxycycline-treated cells. Note that, among the mitochondrial down-regulated proteins by the two treatments, eight are commonly down-regulated. (B) Venn diagram of glycolytic down-regulated proteins in MGT-treated cells versus doxycycline-treated cells. Note that the two different treatments down-regulated several glycolytic proteins and four of those were in common among the two.

Afterwards, quantitative proteomic analysis was performed on protein samples obtained from MCF7 cells treated with MGT (0.2 mg/ml) or with vehicle alone (media). Noticeably, the mass spectrometry analysis validated the results obtained by Seahorse analysis. Indeed, data analysis demonstrated that the expression of 57 mitochondrial-related proteins as well as 6 glycolytic proteins were significantly down-regulated by ≥1.2 fold, after MGT treatment (Fig. 4). Previously, we published that treatment of MCF7 cells with the antibiotic doxycycline significantly inhibited CSCs propagation and decreased the expression of mitochondrial and glycolytic proteins, as assessed by proteomics analysis [29]. Thus, proteomics results of doxycycline-treated MCF7 cells were compared with proteomics results of MGT-treated MCF7 cells. Venn diagram analysis revealed that, among the mitochondrial down-regulated proteins, eight were in common between the two datasets (Fig. 4). Moreover, among the glycolytic down-regulated proteins, four were in common between the two datasets (Fig. 4). Intriguingly, MGT treatment potently induced the down-regulation of mitochondrial and glycolytic proteins, like doxycycline treatment (Table 1A and Table 1B).

**Figure:**
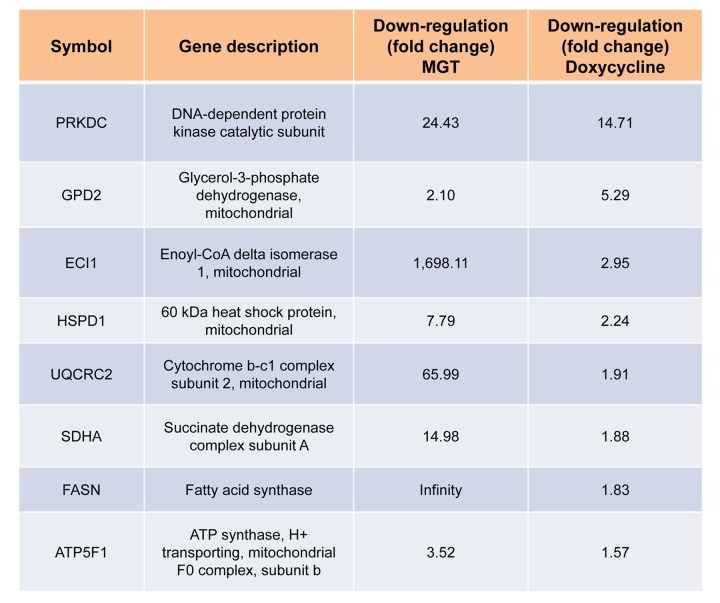
**Table 1A. Commonly down-regulated proteins in MCF7 cells after treatment with doxycycline or MGT.** List of down-regulated mitochondrial proteins and relative fold change.

**Figure:**
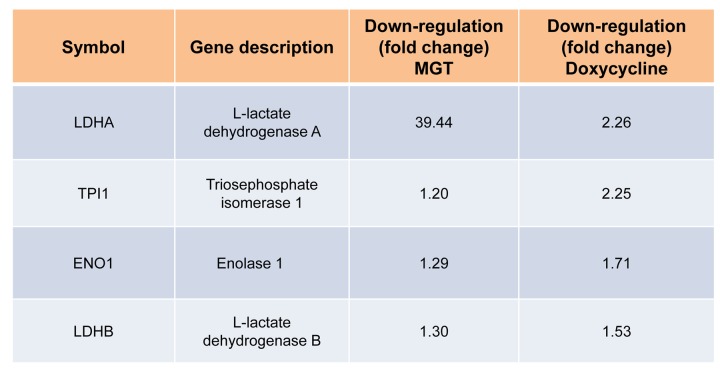
**Table 1B. Commonly down-regulated proteins in MCF7 cells after treatment with doxycycline or MGT.** List of down-regulated glycolytic proteins and relative fold change.

**Figure 5:**
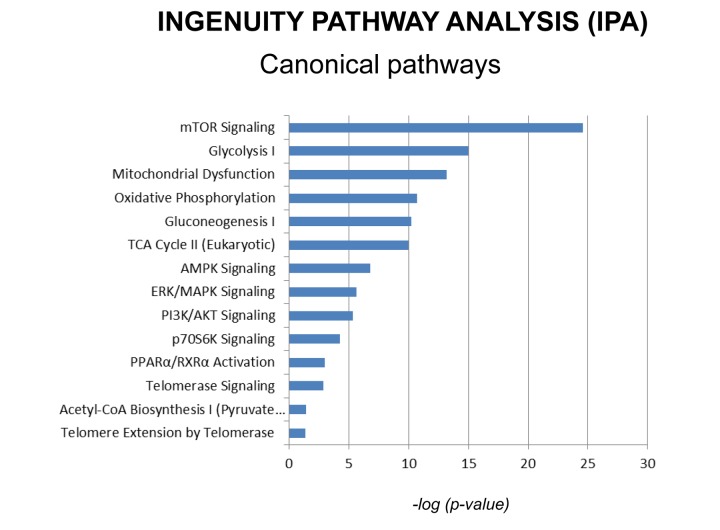
**Canonical pathways affected by MGT in MCF7 cells.** Ingenuity pathways analysis (IPA) showed the cellular pathways most significantly (*p*<0.05) affected by MGT treatment. The *p* value for each pathway is represented with a bar and reported as the negative log of the *p* value.

Additionally, proteomics data were subjected to a bioinformatic analysis with the Ingenuity Pathway Analysis (IPA) software to identify metabolic pathways affected by MGT treatment and deepen our understanding of the altered cellular pathways upon MGT treatment. Fig. 5 depicts the main pathways altered by MGT treatment, which fall under four main areas, as described below.

### Metabolism

**Figure 6:**
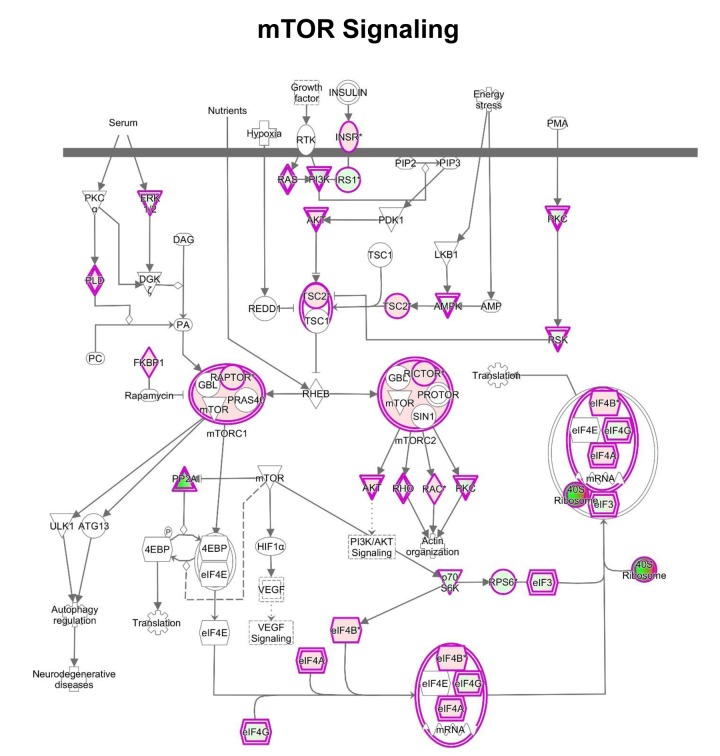
**IPA analysis: Schematic representation of mTOR pathway.** IPA analysis revealed changes in the expression of proteins involved in mTOR signaling after MGT treatment for 48 hours. In this map, the 40S ribosome was indicated as dramatically down-regulated (intense green color), suggesting likely inhibition of protein translation.

One of the pathways mostly altered by MGT treatment is the mTOR signaling pathway, a key regulator of cellular metabolism. Fig. 6 and Supplementary Table S1 show indeed that several genes in the mTOR pathway are differentially expressed in MGT-treated cells compared to control cells. Notably, MGT treatment induces a relevant decrease in numerous proteins of the small ribosomal subunit 40S, with a likely compensatory up-regulation of eukaryotic translation initiation factors. This imbalance may lead to decreased protein synthesis. Moreover, PPP2R5C, a key player in cell proliferation, differentiation, and transformation [30,31], is infinitely down-regulated. PPP2R5C is a regulatory B subunit of protein phosphatase 2A (PP2A), which is one of the main serine-threonine phosphatases in mammalian cells, and it maintains cell homeostasis by counteracting most of the kinase-driven intracellular signaling pathways [32]. Consistent with infinite PPP2R5C down-regulation, our findings demonstrate inhibition of CSCs proliferation upon treatment with MGT.

**Figure 7:**
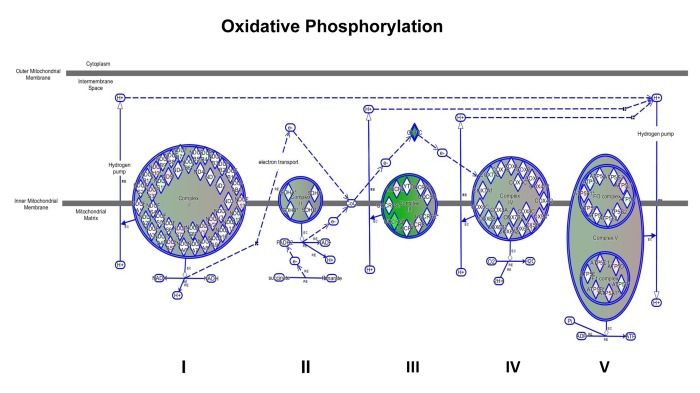
**Impairment of mitochondrial functions uncovered by IPA analysis.** Depicted is the map of oxidative phosphorylation. All the mitochondrial complexes are affected by the treatment, particularly complex III is dramatically down-regulated, as indicated by the intense green colour.

We have shown above that MGT treatment of MCF7 cells decreased mitochondrial respiration, resulting in a reduced basal respiration and ATP production, as well as glycolysis and glycolytic capacity (Figs. 3 and 4). Consistently, IPA analysis confirmed the down-regulation of several proteins involved in all oxidative phosphorylation complexes (Fig. 7). In particular, cytochrome c, which plays a central role in the electron transport chain in the mitochondria, was infinitely down-regulated (Supplementary Table S2). Complex III is particularly affected by the treatment with MGT (Fig. 7). These results are consistent with recently published study from our group, showing that the anti-malaria drug atovaquone, which specifically inhibits mitochondrial complex III, halts the propagation of MCF7-derived CSCs [33]. Finally, the expression of several enzymes in the tricarboxylic acid cycle (TCA) (Supplementary Table S3) and in the mitochondrial dysfunction pathway were found altered (Supplementary Table S4), with a distinct up-regulation of complex V subunits, very likely a compensatory mechanism in response to the MGT-induced inhibition of complex III. Altogether, these data strongly suggest that MGT impairs mitochondrial respiration in MCF7 breast cancer cells.

**Figure 8:**
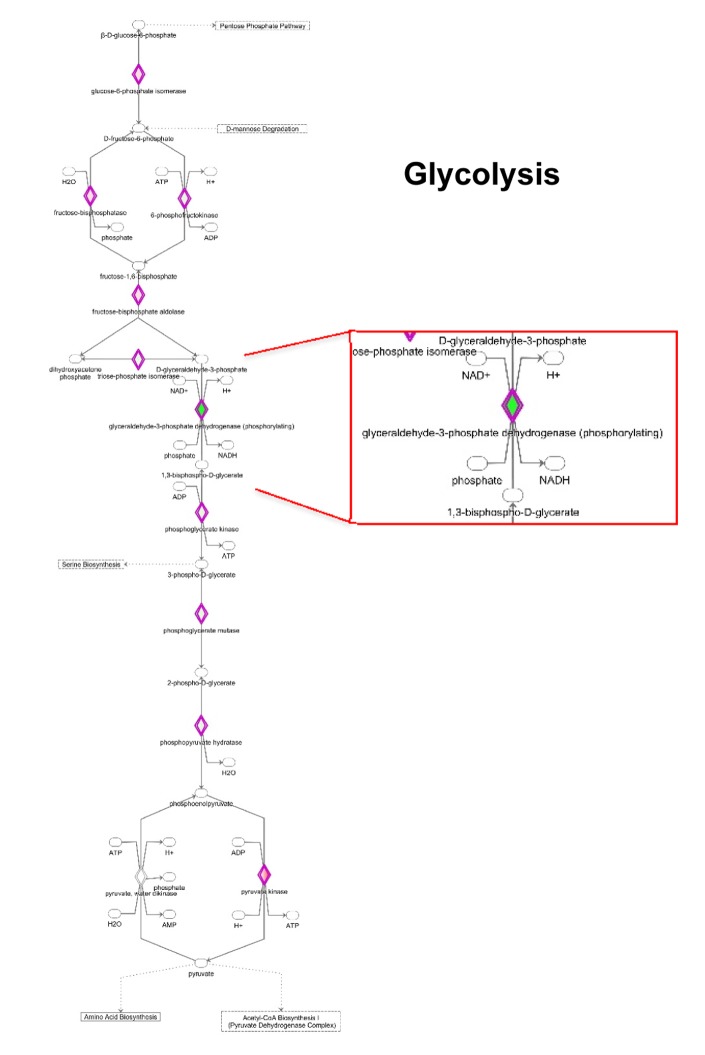
**Modifications in the expression of glycolytic enzymes.** Schematic representation of glucose metabolism upon MGT treatment. Green tea exposure causes an evident impairment of glycolytic pathway. Proteins down-regulated (in green) or up-regulated (in red) are shown.

IPA analysis also revealed significant alterations in the glycolytic pathway (Fig. 8 and Supplementary Table S5). Notably, the expression of some enzymes involved in the initial preparatory phase of glycolysis (glucose conversion to glyceraldehyde 3-phosphate) is affected by MGT treatment. Indeed, enzymes that catalyze reversible reactions, such as aldolase and phosphoglucose isomerase, are up-regulated as compared to untreated cells. However, fructose-bisphosphatase 1 and 2 (FBP1 and 2) are up-regulated by 16 fold after MGT treatment. FBP is a key enzyme of gluconeogenesis, catalyzing the conversion from fructose 1,6- bisphosphate to fructose 6-phosphate, the opposite reaction of phosphofructokinase (PFK), the key regulatory and irreversible step of glycolysis, suggesting that glycolysis might be blocked at the PFK step in MGT-treated cells. Indeed, two PFK isoforms (PFKL and PFKP) are significantly down-regulated after MGT treatment (Supplementary Table S5).

Consistent with this notion, some glycolytic enzymes were dramatically down-regulated by MGT treatment. Phosphoglycerate mutase expression was decreased by 250 fold. Glyceraldehyde 3-phosphate dehydrogenase (GAPDH), which catalyzes the conversion of glyceraldehyde 3-phosphate to 1,3-bisphosphoglycerate, was infinitely down-regulated in MGT-treated cells. Importantly, it was previously shown that vitamin C selectively and potently inhibits glycolysis by targeting GAPDH [34]. We have also shown that vitamin C halts the propagation of highly glycolytic doxycycline-resistant CSCs [35]. Thus, the current results suggest the intriguing possibility that MGT might similarly act as a glycolysis inhibitor, via GAPDH inhibition. Moreover, IPA analysis suggests that there was no production of lactate from pyruvate since key enzymes involved in the fermentation of pyruvate to lactate were remarkably down-regulated (Supplementary Table S5). Altogether with the functional data of Seahorse analysis, these results suggest that MGT effectively inhibits the glycolytic pathway.

**Figure 9:**
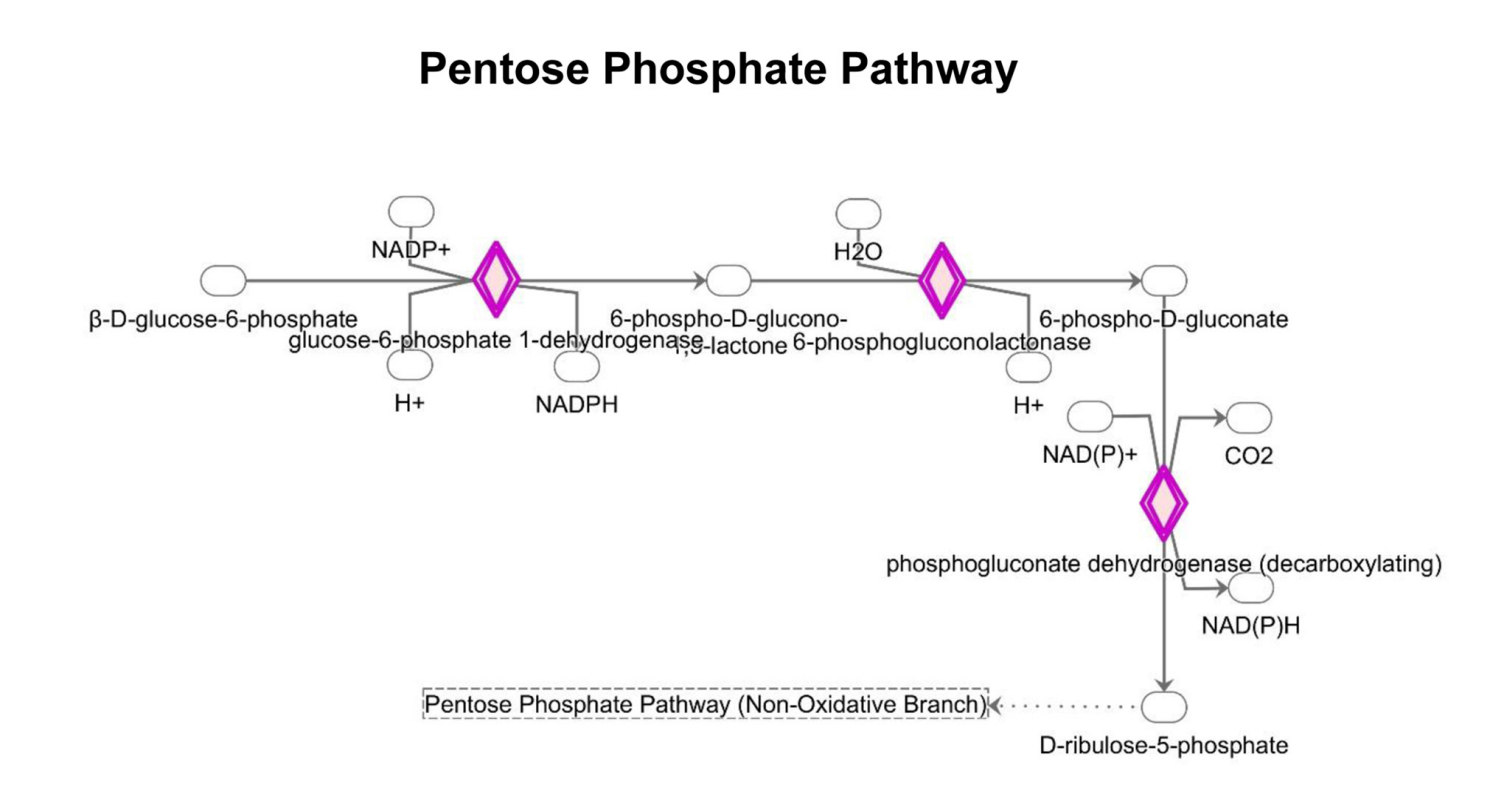
**Changes in the expression of proteins implicated in the pentose phosphate pathway.** MGT treatment up-regulates the expression of proteins involved in the PPP pathway (red color).

Interestingly, the Pentose Phosphate Pathway (PPP) is activated (Fig. 9 and Supplementary Table S6). Tea cathechins may act both as anti-oxidant and as pro-oxidants [36]. As a consequence, upon exposure to MGT, cells may need to regenerate large amounts of the antioxidant cofactor NADPH. In the cytosol, NADPH is normally reduced from NADP+ during the PPP oxidative phase, which serves as a major source of NADPH. Indeed, Supplementary Table S6 shows that the 3 key enzymes of the PPP oxidative phase are upregulated by MGT treatment. MGT causes decreased expression of GAPDH (Supplementary Table S5), likely re-routing the metabolic flux from glycolysis to PPP, to allow the cells to generate more NADPH.

Finally, MGT-treated cells display an increase in the beta-oxidation pathway (Supplementary Table S7), most likely in an attempt to compensate for the decreased mitochondrial respiration and glycolysis. In fact, several enzyme involved in beta-oxidation are elevated, including ECHS1 (enoyl-CoA hydratase), HADH (Hydroxyacyl-CoA dehydrogenase), ACAA (acetyl-CoA acyltransferase), respectively catalyzing the second, third, and fourth step of beta-oxidation. Consistent with an increase in beta-oxidation, the enzymes involved in the Acetyl-CoA biosynthesis (Supplementary Table S8-A) are also increased. Moreover, Supplementary Table S8-B shows that BDH1 (3-hydroxybutyrate dehydrogenase) expression is remarkably increased after MGT treatment. Upon fatty acid degradation, large amounts of acetyl-CoA are generated and in the absence of functional mitochondrial metabolism, acetyl-CoA maybe converted to beta-hydroxybutyrate in a 3-step pathway that critically involves BDH1. Importantly, beta-hydroxyburyrate is regarded as a metabolic marker of lipid oxidation [37,38]. Note that lipid synthesis pathway is likely inhibited, given that one of the most important enzyme, i.e. fatty acid synthase (FASN) was infinitely down-regulated (Supplementary Table S9).

### Cell cycle regulation

**Figure 10:**
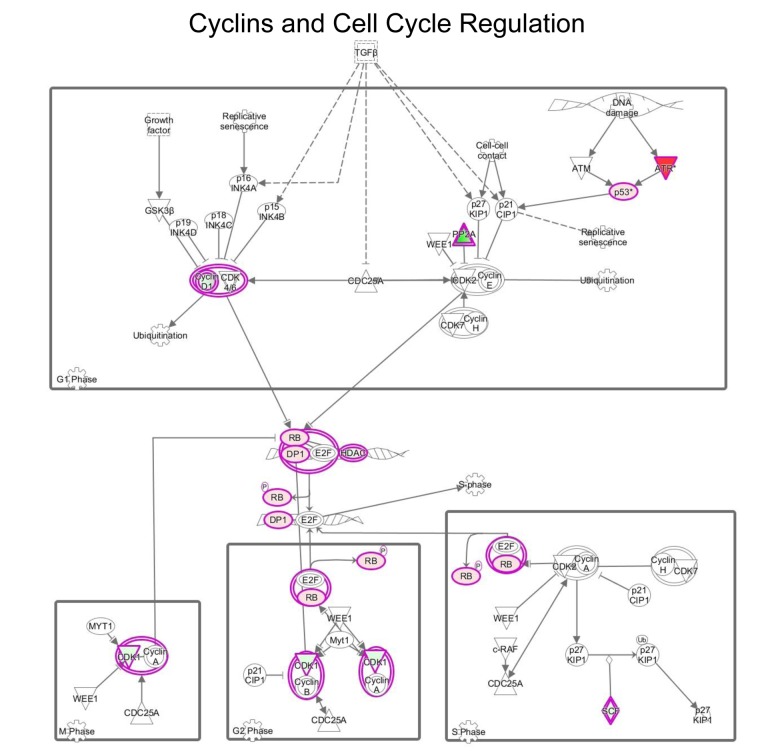
**MGT treatment affects cell cycle regulation in MCF7 cells.** Schematic representation of cell cycle regulation upon treatment with green tea. Proteins down-regulated (in green) or up-regulated (in red) are shown.

IPA analysis of our proteomics data detected alterations in cell cycle regulation after treatment with MGT (Fig. 10 and Supplementary Table S10). The p53 tumour suppressor protein, which is a transcription factor stabilized and activated in response to a range of cellular stresses including hyper-proliferation, DNA damage and shortage of glucose, was significantly up-regulated (by 5.9 fold) as compared to untreated cells (Supplementary Table S10). Phosphorylation of p53 necessary for its activation occurs normally by the action of two proteins, namely ATM and ATR protein kinases. Supplementary Table S10 shows that the ATR protein kinase is infinitely up-regulated. This finding suggests that MGT may promote a p53-induced cell-cycle arrest or programmed cell death. Furthermore, the tumor suppressor retinoblastoma protein (RB1) is significantly up-regulated by MGT treatment, likely inhibiting cell cycle progression (Supplementary Table S10). In addition, it is well known that the RB1 attracts histone deacetylase 1 (HDAC1) to the chromatin, reducing transcription of S-phase promoting factors, further suppressing DNA synthesis [39]. Consistent with this notion, Supplementary Table S10 shows the up-regulation HDAC1, upon MGT treatment. Cyclins/CDKs-mediated hyper-phosphorylation of RB1 serves to inactivate RB1, allowing cell cycle progression. Interestingly, MGT treatment reduces the levels of cyclins /CDKs, presumably activating RB1 and hindering the progression of cell cycle (Supplementary Table S10). Lastly, MGT treatment down-regulates by 18-fold SKP1 (S-phase kinase-associated protein 1), a protein involved in the ubiquitination of cell cycle proteins (Supplementary Table S10). Although the role of this protein during tumorigenesis remains unclear, it is known that SKP complex targets CDK inhibitor p27 for degradation [40] and it has been recently published that in non-small cell lung cancers elevated levels of SKP1 was associated with poor prognosis [41]. Altogether, these data suggest that MGT inhibits cells cycle progression by up-regulation of p53 and RB1. These data are consistent with previously published findings showing that green tea polyphenols display anti-tumors activities of MCF7 cells, by inducing cell cycle arrest and mitochondrial-mediated apoptosis [42].

### Antioxidant response

This study demonstrates that treatment with MGT up-regulates the enzymes of the oxidative branch of the pentose phosphate pathway, which is responsible for the production of the antioxidant NADPH (Fig. 9 and Supplementary Table S6). Moreover, IPA analysis revealed that several proteins implicated in the NRF2-stress-related pathway were significantly altered (Supplementary Table S11). In particular, note the increase of glutathione S transferases, enzymes involved in detoxification processes. Similarly, catalase, thioredoxin, NQO1 and superoxide dismutase, which all play important roles in anti-oxidant responses [43,44], were all found up-regulated after MGT treatment.

### Interleukin pathways

IPA analysis showed that treatment with MGT affected the IL-8 pathway. IL-8 signaling is involved in angiogenesis, proliferation and increment of the migratory capacity of cancer cells. A decrease in the expression of most of the proteins involved in the pathway is observed after MGT treatment (Supplementary Table S12). Interestingly, VCAM 1 and the transcription factor NF-kB (encoded by the RELA gene) are both down-regulated. NF-kB is implicated in many cellular processes, including inflammation, metabolism and chemotaxis, and VCAM 1 mediates pro-metastatic tumor-stromal interactions [45]. These findings suggest that MGT may inhibit: i) IL-8-mediated induction of VCAM1 by blocking ERK1/2 (note that many MAPKs are down-regulated); and ii) NF-kB signaling pathway, leading to a reduction of the inflammatory process, invasion and metastasis.

Moreover, the IPA analysis suggests an impairment of the PI3K/Akt signaling pathway. Note that PIK3C2B (a PI3K family member) is down-regulated by 8.49 fold, likely leading to impaired phosphorylation and activation of AKT and promoting pro-apoptotic events. Consistent with this notion, the expression of the apoptosis regulator BAX protein expression is increased.

## DISCUSSION

**Figure 11:**
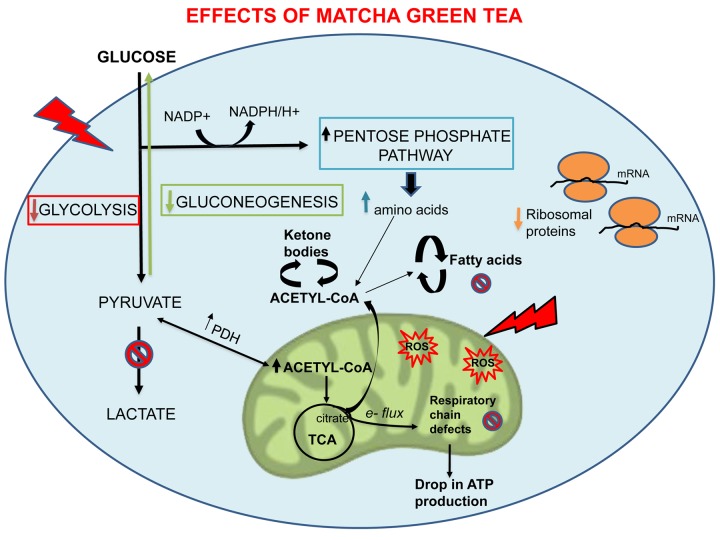
**Overview of the metabolic and cellular processes affected by MGT.** Note that MGT treatment inhibits cellular metabolism (glycolysis, mitochondrial respiration, fatty acid synthesis) with a likely compensatory increase in fatty acid breakdown.

Over the past years, the anti-cancer properties of green tea or its components have been investigated. Its effectiveness is reported across several types of tumor. Importantly, previous studies have reported that green tea and epigallocatechin gallate (EGCG), one of the main components of green tea, suppresses cancer stem-like cells (CSCs) properties in a variety of cellular models [46-48]. Here, we aimed to investigate if the inhibitory efficacy of MGT on CSCs is associated with inhibition of cellular metabolism. The results presented in this paper demonstrate that MGT preferentially inhibits CSC proliferative expansion derived from MCF7 breast cancer cells. Furthermore, MGT treatment overall reduced cellular metabolism, including mitochondrial respiration and glycolysis, as assessed by Seahorse analysis. These phenotypic results were integrated with proteomic analysis data obtained from MGT-treated cells. Bioinformatics analysis through Ingenuity Pathway Analysis (IPA) showed that MGT not only affected mitochondrial respiration and glycolysis, but also fatty acid synthesis, validating results already described in the literature. It has been reported that EGCG reduces *in vivo* the expression of several hepatic enzymes, including fatty acid synthase (FASN) [49]. FASN is an enzyme overexpressed in many cancer types that plays a key role in tumorigenesis [50]. Consistently, our results demonstrate that MGT treatment dramatically decreased FASN expression, as compared to control cells. Furthermore, we also found that gluconeogenesis was down-regulated by MGT treatment, concordantly with *in vivo* and *in vitro* results on human colon carcinoma Caco-2 cells treated with EGCG [51]. The metabolic changes observed in MCF7 upon treatment with MGT are summarized in Fig. 11.

IPA analysis indicates that MGT may have broad and profound effects on a variety of cellular processes. Indeed, our findings suggest that MGT may affect cell cycle progression and the oxidative stress response in MCF7 cells. These effects have been already described in different cancer types [52,53]. Furthermore, MGT treatment may decrease inflammation and angiogenesis, as well as may inhibit the mTOR pathway (target of rapamycin), impairing protein synthesis. This raises the intriguing possibility that the natural compound MGT can be used as inhibitor of mTOR, instead of chemical compounds, such as rapamycin.

Lastly, analysis of our proteomic data revealed that MGT displays overlapping effects in targeting mitochondria with the antibiotic doxycycline. Thus, the natural compound MGT may help prevent tumor recurrence and metastases, reducing the side effects of the anti-cancer therapy, similarly to what has been proposed for doxycycline [54].

## CONCLUSIONS

In summary, we found that the natural compound Matcha green tea mechanistically targets oxidative phosphorylation and therefore CSC propagation. Importantly, we demonstrated that MGT effectively down-regulated oxygen consumption rate (OCR) and extracellular acidification rate (ECAR). Moreover, MGT treatment impaired others cellular metabolic pathways, and several cell signaling pathways such as cell cycle regulation, antioxidant response and inflammation. Altogether, these findings strongly propose MGT as a natural compound that may help to overcome cancer cell resistance to chemotherapy. Surely, more molecular studies and clinical trials are warranted.

## MATERIALS AND METHODS

### Materials

MCF7 were purchased from ATCC. Media for cell cultures was DMEM (D6546, Sigma-Aldrich). Cell culture media (DMEM/F12) for spheroid culture was purchased from Life Technologies. Sulforhodamine B (SRB), 1x Trypsin-EDTA, 2-hydroxyethylmethacrylate (poly-HEMA) were purchased from Sigma-Aldrich. Matcha green tea (MGT) powder was a generous gift from OMGTea, Inc. (UK). The exact chemical composition of the product is available upon request from the manufacturer [Contact Katherine Swift (Founder, OMGTea); Email: katherine@omgteas.co.uk].

### Tumour-spheroids culture

A single cell suspension was prepared using enzymatic (1x Trypsin-EDTA) and manual disaggregation (25 gauge needle) [55]. Five thousand cells were plated with spheroids medium (DMEM-F12/B27/EGF-20ng/ml/PenStrep), in non-adherent conditions, in six wells plates coated with poly-HEMA. After five days of culture, the number of spheres with diameter >50 µm per well were counted.

### Seahorse analysis

Extracellular acidification rates (ECAR) and oxygen consumption rates (OCR) were analysed using the Seahorse XFe96 bioenergetic analyser (Seahorse Bioscience, MA, USA). Six thousand MCF7 cells were seeded per well, into XF96-well cell culture plates, and incubated at 37°C in a 5% CO2 humidified atmosphere. MCF7 cells were maintained in DMEM supplemented with 10% FBS (Fetal Bovine Serum), 2 mM GlutaMAX, and 1% Pen- Strep. Twenty-four hours from the plating, cells were incubated with/without filtered MGT at the concentration of 0.2 mg/ml (dissolved in water). After seventy-two hours, cells were washed in pre-warmed XF assay media as previously described [56]. ECAR and OCR measurements were normalized by cellular protein content (SRB). Data set was analyzed by XFe-96 software and Excel software, using Student’s t-test calculations. All experiments were performed in sextuplicate, three times independently.

### SRB assay

Cells were subjected to SRB colorimetric assay for cytotoxicity, based on the measurement of cellular protein content. Briefly, MCF7 cells in monolayers were first fixed with 10% trichloroacetic acid and then washed with 1% acetic acid. The OD determination at 510 nm using a microplate reader was done after the dye dissolution in 10mMTris base solution [57].

### Quantitative proteomic analysis

Cell lysates, obtained after treatment with MGT for 48 hours, were subjected to quantitative proteomic analysis as previously described [29]. Data were analyzed using the Mascot search engine. Five technical replicates were made for each sample (untreated and MGT-treated cells). Statistical analyses were performed using ANOVA and only fold-changes in proteins greater than 1.2, with a p-value less than 0.05 were considered significant.

### Ingenuity pathway analyses

Ingenuity Pathway Analysis (IPA) (Ingenuity systems, http://www.ingenuity.com) was employed to analyze pathways and functions in the proteomics datasets. IPA aids the interpretation of the proteomics data, by organizing differentially expressed proteins into noted functions and pathways.

### Statistics

All data are presented as means ± SEM. Student’s t test was used. P < 0.05 was considered statistically significant.

## Supplementary Material

OMGTea's Organic Matcha Green Tea Nutritional Analysis

Supplementary Tables
