# Supplementary material for: Matcha green tea (MGT) inhibits the propagation of cancer stem cells (CSCs), by targeting mitochondrial metabolism, glycolysis and multiple cell signalling pathways
Source: Aging (Albany NY). 2018 Aug 23;10(8):1867–83. doi: 10.18632/aging.101483 (PMC6128439; doi:10.18632/aging.101483)
Supplement: OMGTea's Organic Matcha Green Tea Nutritional Analysis [file aging-10-101483-s001.pdf]

OMGTea's Organic Matcha Green Tea Nutritional Analysis
Per Serving (1 gram)

**Grade AAA (100% matcha)**	
**Serving Size: 1g**	
**Servings Per tin: 30**	
	**Amount Per Serving**
Total Calories	3,18 Kcal/g
	13,33 KJ/g
Calories from Fat	0,37 Kcal/g
Carbohydrate	206 mg/g
Sugars	15,9 mg/g
Total Dietary Fibers	331 mg/g
Proteins	334 mg/g
Total Fat	41 mg/g
Saturated Fatty Acid	12 mg/g
Monounsaturated Fatty Acid	4,8 mg/g
Polyunsaturated Fatty Acid	24 mg/g
Trans Fatty Acid	0,29 mg/g
ORAC Score	2140-3170 unit
EGCg	67 mg/g
Caffeine	32 mg/g
L-Theanine	12,37 mg/g
Ash	63 mg/g
Sodium	0,11 mg/g
