# Supplementary material for: Matcha green tea (MGT) inhibits the propagation of cancer stem cells (CSCs), by targeting mitochondrial metabolism, glycolysis and multiple cell signalling pathways
Source: Aging (Albany NY). 2018 Aug 23;10(8):1867–83. doi: 10.18632/aging.101483 (PMC6128439; doi:10.18632/aging.101483)
Supplement: Supplementary Tables [file aging-10-101483-s002.pdf]

Table S1

mTOR Signaling

Symbol	Entrez Gene Name	Fold Change
AKT1	AKT serine/threonine kinase 1	1.771
EIF3A	eukaryotic translation initiation factor 3 subunit A	7.762
EIF3B	eukaryotic translation initiation factor 3 subunit B	-22.64
EIF3C	eukaryotic translation initiation factor 3 subunit C	4.348
EIF3D	eukaryotic translation initiation factor 3 subunit D	2.145
EIF3E	eukaryotic translation initiation factor 3 subunit E	60.409
EIF3F	eukaryotic translation initiation factor 3 subunit F	-1.994
EIF3G	eukaryotic translation initiation factor 3 subunit G	1.263
EIF3H	eukaryotic translation initiation factor 3 subunit H	-3.458
EIF3I	eukaryotic translation initiation factor 3 subunit I	241.866
EIF3J	eukaryotic translation initiation factor 3 subunit J	1.592
EIF3K	eukaryotic translation initiation factor 3 subunit K	1.649
EIF3L	eukaryotic translation initiation factor 3 subunit L	11.723
EIF3M	eukaryotic translation initiation factor 3 subunit M	-1.737
EIF4A1	eukaryotic translation initiation factor 4A1	1.924
EIF4A2	eukaryotic translation initiation factor 4A2	1.924
EIF4A3	eukaryotic translation initiation factor 4A3	44.568
EIF4B	eukaryotic translation initiation factor 4B	2.191
EIF4G1	eukaryotic translation initiation factor 4 gamma 1	-1.688
EIF4G2	eukaryotic translation initiation factor 4 gamma 2	-1.688
EIF4G3	eukaryotic translation initiation factor 4 gamma 3	11.626
FKBP1A	FK506 binding protein 1A	1.312
INSR	insulin receptor	5.4
IRS1	insulin receptor substrate 1	-29.26
KRAS	KRAS proto-oncogene, GTPase	10.706
MAPK3	mitogen-activated protein kinase 3	-2.007
NRAS	neuroblastoma RAS viral oncogene homolog	-1.867
PIK3C2B	phosphatidylinositol-4-phosphate 3-kinase catalytic subu	-8.49
PLD3	phospholipase D family member 3	1.565
PPP2CA	protein phosphatase 2 catalytic subunit alpha	2.682

Table S1 (continued)

PPP2CB	protein phosphatase 2 catalytic subunit beta	-1.25
PPP2R1A	protein phosphatase 2 scaffold subunit Aalpha	4.588
PPP2R1B	protein phosphatase 2 scaffold subunit Abeta	18.873
PPP2R2B	protein phosphatase 2 regulatory subunit Bbeta	-3.977
PPP2R5C	protein phosphatase 2 regulatory subunit B'gamma	Infinity (Down)
PPP2R5D	protein phosphatase 2 regulatory subunit B'delta	-2.002
PPP2R5E	protein phosphatase 2 regulatory subunit B'epsilon	3.593
PRKAA1	protein kinase AMP-activated catalytic subunit alpha 1	-1.241
PRKAG1	protein kinase AMP-activated non-catalytic subunit gam	1.245
PRKCD	protein kinase C delta	-4.107
PRKD3	protein kinase D3	-1.229
PTPA	protein phosphatase 2 phosphatase activator	6.214
RAC1	ras-related C3 botulinum toxin substrate 1 (rho family, sm	2.185
RHOA	ras homolog family member A	-2.213
RHOB	ras homolog family member B	1.505
RHOC	ras homolog family member C	-1.371
RHOG	ras homolog family member G	1.386
RHOQ	ras homolog family member Q	-1.253
RHOT2	ras homolog family member T2	-1.304
RICTOR	RPTOR independent companion of MTOR complex 2	2.262
RPS2	ribosomal protein S2	-1.991
RPS3	ribosomal protein S3	-2.982
RPS5	ribosomal protein S5	-4373.001
RPS6	ribosomal protein S6	-1.669
RPS7	ribosomal protein S7	Infinity (Down)
RPS8	ribosomal protein S8	-389.59
RPS9	ribosomal protein S9	162.413
RPS10	ribosomal protein S10	1.701
RPS11	ribosomal protein S11	4040.875

Table S1 (continued)

RPS12	ribosomal protein S12	-1.769
RPS13	ribosomal protein S13	1.833
RPS14	ribosomal protein S14	5.41
RPS15	ribosomal protein S15	34.906
RPS16	ribosomal protein S16	2.508
RPS17	ribosomal protein S17	12.002
RPS18	ribosomal protein S18	-311.164
RPS19	ribosomal protein S19	-5.031
RPS20	ribosomal protein S20	2.155
RPS21	ribosomal protein S21	-1.486
RPS23	ribosomal protein S23	4.441
RPS24	ribosomal protein S24	1.381
RPS25	ribosomal protein S25	2.474
RPS27	ribosomal protein S27	-1.244
RPS28	ribosomal protein S28	-7.32
RPS29	ribosomal protein S29	Infinity (Up)
RPS15A	ribosomal protein S15a	41.315
RPS27A	ribosomal protein S27a	-1.41
RPS3A	ribosomal protein S3A	-91.728
RPS4X	ribosomal protein S4, X-linked	-18.135
RPS4Y1	ribosomal protein S4, Y-linked 1	-20.486
RPS6KA1	ribosomal protein S6 kinase A1	2.045
RPS6KA3	ribosomal protein S6 kinase A3	-1.917
RPS6KB1	ribosomal protein S6 kinase B1	-1.302
RPSA	ribosomal protein SA	2.22
RPTOR	regulatory associated protein of MTOR complex 1	3.475
TSC2	tuberous sclerosis 2	1.784

Table S2

Oxidative Phosphorylation

Symbol	Entrez Gene Name	Fold Change
COX6B1	cytochrome c oxidase subunit 6B1	-1.209
COX7A2	cytochrome c oxidase subunit 7A2	-1.315
COX7A2L	cytochrome c oxidase subunit 7A2 like	-1.297
CYC1	cytochrome c1	-12.856
CYCS	cytochrome c, somatic	Infinity (Down)
NDUFA2	NADH:ubiquinone oxidoreductase subunit A2	-1.433
NDUFA4	NDUFA4, mitochondrial complex associated	-2.049
NDUFA12	NADH:ubiquinone oxidoreductase subunit A12	-1.479
NDUFB4	NADH:ubiquinone oxidoreductase subunit B4	-1.372
NDUFB6	NADH:ubiquinone oxidoreductase subunit B6	-1.413
NDUFB7	NADH:ubiquinone oxidoreductase subunit B7	-1.315
NDUFS1	NADH:ubiquinone oxidoreductase core subunit S1	-9.407
NDUFS8	NADH:ubiquinone oxidoreductase core subunit S8	-222.002
SDHA	succinate dehydrogenase complex flavoprotein subunit A	-14.978

Table S3

TCA Cycle

Symbol	Entrez Gene Name	Fold Change
ACO2	aconitase 2	-2.079
CS	citrate synthase	1.306
DHTKD1	dehydrogenase E1 and transketolase domain containing 1	-32.649
DLD	dihydrolipoamide dehydrogenase	Infinity (Up)
DLST	dihydrolipoamide S-succinyltransferase	-6.29
FH	fumarate hydratase	5.42
IDH3A	isocitrate dehydrogenase 3 (NAD(+)) alpha	-1.916
IDH3B	isocitrate dehydrogenase 3 (NAD(+)) beta	-2.04
IDH3G	isocitrate dehydrogenase 3 (NAD(+)) gamma	2.21
MDH1	malate dehydrogenase 1	8.094
MDH2	malate dehydrogenase 2	1.334
OGDH	oxoglutarate dehydrogenase	2.951
OGDHL	oxoglutarate dehydrogenase-like	-2.862
SDHA	succinate dehydrogenase complex flavoprotein subunit A	-14.978
SDHB	succinate dehydrogenase complex iron sulfur subunit B	-1.45
SUCLA2	succinate-CoA ligase ADP-forming beta subunit	2.055
SUCLG1	succinate-CoA ligase alpha subunit	2.374

Table S4

Mitochondrial Dysfunction

Symbol	Entrez Gene Name	Fold Change
ACO2	aconitase 2	-2.079
AIFM1	apoptosis inducing factor, mitochondria associated 1	1.797
ATP5A1	ATP synthase, H+ transporting, mitochondrial F1 complex, alpha subunit 1, cardiac muscle	1.473
ATP5B	ATP synthase, H+ transporting, mitochondrial F1 complex, beta polypeptide	4.632
ATP5C1	ATP synthase, H+ transporting, mitochondrial F1 complex, gamma polypeptide 1	2.57
ATP5D	ATP synthase, H+ transporting, mitochondrial F1 complex, delta subunit	1.203
ATP5F1	ATP synthase, H+ transporting, mitochondrial Fo complex subunit B1	12.921
ATP5H	ATP synthase, H+ transporting, mitochondrial Fo complex subunit D	-1.195
ATP5I	ATP synthase, H+ transporting, mitochondrial Fo complex subunit E	3.237
ATP5J	ATP synthase, H+ transporting, mitochondrial Fo complex subunit F6	-2.558
ATP5L	ATP synthase, H+ transporting, mitochondrial Fo complex subunit G	35.795
ATP5O	ATP synthase, H+ transporting, mitochondrial F1 complex, O subunit	-2.561
CAT	catalase	6.285
COX4I1	cytochrome c oxidase subunit 4I1	1.23
COX5A	cytochrome c oxidase subunit 5A	30.335
COX5B	cytochrome c oxidase subunit 5B	1.471
COX6B1	cytochrome c oxidase subunit 6B1	-1.209
COX7A2	cytochrome c oxidase subunit 7A2	-1.315
COX7A2L	cytochrome c oxidase subunit 7A2 like	-1.297
CPT1A	carnitine palmitoyltransferase 1A	-6.836
CYB5A	cytochrome b5 type A	1.424
CYB5R3	cytochrome b5 reductase 3	4.597
CYC1	cytochrome c1	-12.856
CYCS	cytochrome c, somatic	Infinity (Down)
GPD2	glycerol-3-phosphate dehydrogenase 2	7.706
GSR	glutathione-disulfide reductase	1.57
HSD17B10	hydroxysteroid 17-beta dehydrogenase 10	10.155
LRRK2	leucine rich repeat kinase 2	-26.533
MT-ND5	NADH dehydrogenase, subunit 5 (complex I)	1.542
NDUFA2	NADH:ubiquinone oxidoreductase subunit A2	-1.433

Table S4 (continued)

NDUFA4	NDUFA4, mitochondrial complex associated	-2.049
NDUFA5	NADH:ubiquinone oxidoreductase subunit A5	2.673
NDUFA12	NADH:ubiquinone oxidoreductase subunit A12	-1.479
NDUFA13	NADH:ubiquinone oxidoreductase subunit A13	5.724
NDUFB4	NADH:ubiquinone oxidoreductase subunit B4	-1.372
NDUFB6	NADH:ubiquinone oxidoreductase subunit B6	-1.413
NDUFB7	NADH:ubiquinone oxidoreductase subunit B7	-1.315
NDUFB10	NADH:ubiquinone oxidoreductase subunit B10	3.613
NDUFS1	NADH:ubiquinone oxidoreductase core subunit S1	-9.407
NDUFS3	NADH:ubiquinone oxidoreductase core subunit S3	18.873
NDUFS7	NADH:ubiquinone oxidoreductase core subunit S7	1.443
NDUFS8	NADH:ubiquinone oxidoreductase core subunit S8	-222.002
NDUFV1	NADH:ubiquinone oxidoreductase core subunit V1	1.547
NDUFV2	NADH:ubiquinone oxidoreductase core subunit V2	6.795
OGDH	oxoglutarate dehydrogenase	2.951
PARK7	Parkinsonism associated deglycase	7.048
PDHA1	pyruvate dehydrogenase (lipoamide) alpha 1	2.472
PRDX3	peroxiredoxin 3	2.963
PRDX5	peroxiredoxin 5	1.31
RHOT2	ras homolog family member T2	-1.304
SDHA	succinate dehydrogenase complex flavoprotein subunit A	-14.978
SDHB	succinate dehydrogenase complex iron sulfur subunit B	-1.45
TXNRD2	thioredoxin reductase 2	12.611
UQCRB	ubiquinol-cytochrome c reductase binding protein	2.006
UQCRC1	ubiquinol-cytochrome c reductase core protein I	12.438
UQCRC2	ubiquinol-cytochrome c reductase core protein II	-65.988
UQCRFS1	ubiquinol-cytochrome c reductase, Rieske iron-sulfur polypeptide 1	-4.501
UQCRQ	ubiquinol-cytochrome c reductase complex III subunit VII	Infinity (Down)
VDAC1	voltage dependent anion channel 1	9.124
VDAC2	voltage dependent anion channel 2	1.42
VDAC3	voltage dependent anion channel 3	2.166

Table S5

Glycolysis

Symbol	Entrez Gene Name	Fold Change
ALDOA	aldolase, fructose-bisphosphate A	117.268
ALDOC	aldolase, fructose-bisphosphate C	117.268
ENO1	enolase 1	8.342
ENO2	enolase 2	2.39
ENO3	enolase 3	8.177
ENO4	enolase family member 4	-5.202
FBP1	fructose-bisphosphatase 1	16.879
FBP2	fructose-bisphosphatase 2	16.879
GAPDH	glyceraldehyde-3-phosphate dehydrogenase	Infinity (Down)
GPI	glucose-6-phosphate isomerase	16.48
PFKL	phosphofructokinase, liver type	-2.101
PFKM	phosphofructokinase, muscle	1.914
PFKP	phosphofructokinase, platelet	-27.581
PGAM1	phosphoglycerate mutase 1	-260.514
PGAM2	phosphoglycerate mutase 2	-260.514
PGAM4	phosphoglycerate mutase family member 4	1.601
PGK1	phosphoglycerate kinase 1	35.978
PGK2	phosphoglycerate kinase 2	-58.553
PKLR	pyruvate kinase, liver and RBC	1.637
PKM	pyruvate kinase, muscle	Infinity (Up)
TPI1	triosephosphate isomerase 1	-2.674

Pyruvate fermentation to lactate

Symbol	Entrez Gene Name	Fold Change
LDHA	lactate dehydrogenase A	-39.441
LDHAL6B	lactate dehydrogenase A like 6B	-150.724
LDHB	lactate dehydrogenase B	-1.305

Table S6

Pentose Phosphate Pathway

Symbol	Entrez Gene Name	Fold Change
G6PD	glucose-6-phosphate dehydrogenase	1370.288
PGD	phosphogluconate dehydrogenase	13.995
PGLS	6-phosphogluconolactonase	1.384

Table S7

Fatty Acids β-Oxidation

Symbol	Entrez Gene Name	Fold Change
ACAA1	acetyl-CoA acyltransferase 1	1.51
ACAA2	acetyl-CoA acyltransferase 2	1.922
ECHS1	enoyl-CoA hydratase, short chain 1	Infinity (Up)
ECI1	enoyl-CoA delta isomerase 1	-1698.109
HADH	hydroxyacyl-CoA dehydrogenase	1.579
HADHA	hydroxyacyl-CoA dehydrogenase/3-ketoacyl-CoA thiolase/enoyl-CoA	2.227
HADHB	hydroxyacyl-CoA dehydrogenase/3-ketoacyl-CoA thiolase/enoyl-CoA	-4.459
HSD17B4	hydroxysteroid 17-beta dehydrogenase 4	32.84
HSD17B10	hydroxysteroid 17-beta dehydrogenase 10	10.155
SCP2	sterol carrier protein 2	Infinity (Down)
SLC27A3	solute carrier family 27 member 3	1.266
SLC27A4	solute carrier family 27 member 4	2.475

Table S8

A Pyruvate dehydrogenase complex – Acetyl-CoA biosynthesis I

Symbol	Entrez Gene Name	Fold Change
DLAT	dihydrolipoamide S-acetyltransferase	16.78
DLD	dihydrolipoamide dehydrogenase	Infinity (Up)
PDHA1	pyruvate dehydrogenase (lipoamide) alpha 1	2.472

B Ketolysis

Symbol	Entrez Gene Name	Fold Change
ACAT1	acetyl-CoA acetyltransferase 1	-4.101
ACAT2	acetyl-CoA acetyltransferase 2	-1.912
BDH1	3-hydroxybutyrate dehydrogenase, type 1	151.046
HADHA	hydroxyacyl-CoA dehydrogenase/3-ketoacyl-CoA thiolase/enoyl-CoA hydratase	2.227
HADHB	hydroxyacyl-CoA dehydrogenase/3-ketoacyl-CoA thiolase/enoyl-CoA hydratase	-4.459
OXCT1	3-oxoacid CoA-transferase 1	2.268

Table S9

AMPK Signaling

Symbol	Entrez Gene Name	Fold Change
ACTB	actin beta	511.406
ADRB3	adrenoceptor beta 3	2.198
AK1	adenylate kinase 1	-3.977
AK2	adenylate kinase 2	6.354
AK3	adenylate kinase 3	4.043
AKT1	AKT serine/threonine kinase 1	1.771
ARID2	AT-rich interaction domain 2	10.67
CAB39	calcium binding protein 39	-18.94
CCND1	cyclin D1	-1.822
CPT1A	carnitine palmitoyltransferase 1A	-6.836
CREBBP	CREB binding protein	-9.521
EEF2	eukaryotic translation elongation factor 2	Infinity (Up)
ELAVL1	ELAV like RNA binding protein 1	-1.274
FASN	fatty acid synthase	Infinity (Down)
FOXO6	forkhead box O6	4.363
GNAS	GNAS complex locus	56
INSR	insulin receptor	5.4
IRS1	insulin receptor substrate 1	-29.26
MAPK14	mitogen-activated protein kinase 14	-1.731
PCK2	phosphoenolpyruvate carboxykinase 2, mitochondrial	13.47
PFKL	phosphofructokinase, liver type	-2.101
PFKM	phosphofructokinase, muscle	1.914
PFKP	phosphofructokinase, platelet	-27.581
PIK3C2B	phosphatidylinositol-4-phosphate 3-kinase catalytic subunit type 2 beta	-8.49
PPAT	phosphoribosyl pyrophosphate amidotransferase	-5.531
PPM1A	protein phosphatase, Mg2+/Mn2+ dependent 1A	2.215
PPM1B	protein phosphatase, Mg2+/Mn2+ dependent 1B	2.215

Table S10

Cyclins and Cell Cycle Regulation

Symbol	Entrez Gene Name	Fold Change
ATR	ATR serine/threonine kinase	Infinity (Up)
CCND1	cyclin D1	-1.822
CCND2	cyclin D2	-2.186
CDK1	cyclin dependent kinase 1	-1.575
HDAC1	histone deacetylase 1	4.625
HDAC2	histone deacetylase 2	-1.548
PA2G4	proliferation-associated 2G4	-1.901
PPP2CA	protein phosphatase 2 catalytic subunit alpha	2.682
PPP2CB	protein phosphatase 2 catalytic subunit beta	-1.25
PPP2R1A	protein phosphatase 2 scaffold subunit Aalpha	4.588
PPP2R1B	protein phosphatase 2 scaffold subunit Abeta	18.873
PPP2R2B	protein phosphatase 2 regulatory subunit Bbeta	-3.977
PPP2R5C	protein phosphatase 2 regulatory subunit B'gamma	Infinity (Down)
PPP2R5D	protein phosphatase 2 regulatory subunit B'delta	-2.002
PPP2R5E	protein phosphatase 2 regulatory subunit B'epsilon	3.593
PTPA	protein phosphatase 2 phosphatase activator	6.214
RB1	RB transcriptional corepressor 1	3.123
SKP1	S-phase kinase-associated protein 1	-18.472
TFDP1	transcription factor Dp-1	1.473
TP53	tumor protein p53	5.925

Table S11

NRF2-Mediated Antioxidant Response

Symbol	Entrez Gene Name	Fold Change
ABCC1	ATP binding cassette subfamily C member 1	77.053
ACTA2	actin, alpha 2, smooth muscle, aorta	511.406
ACTB	actin beta	511.406
ACTC1	actin, alpha, cardiac muscle 1	511.406
ACTG2	actin, gamma 2, smooth muscle, enteric	511.406
AKR7A2	aldo-keto reductase family 7 member A2	10.398
AKR7A3	aldo-keto reductase family 7 member A3	-1.554
AKT1	AKT serine/threonine kinase 1	1.771
CAT	catalase	6.285
CBR1	carbonyl reductase 1	1.245
CCT7	chaperonin containing TCP1 subunit 7	-2.906
CREBBP	CREB binding protein	-9.521
DNAJA1	DnaJ heat shock protein family (Hsp40) member A1	4.394
DNAJA3	DnaJ heat shock protein family (Hsp40) member A3	1.706
DNAJB11	DnaJ heat shock protein family (Hsp40) member B11	-1.77
DNAJC1	DnaJ heat shock protein family (Hsp40) member C1	1.726
DNAJC6	DnaJ heat shock protein family (Hsp40) member C6	-3.339
DNAJC8	DnaJ heat shock protein family (Hsp40) member C8	-2.842
DNAJC11	DnaJ heat shock protein family (Hsp40) member C11	2.403
DNAJC17	DnaJ heat shock protein family (Hsp40) member C17	1.687
DNAJC19	DnaJ heat shock protein family (Hsp40) member C19	15.957
EPHX1	epoxide hydrolase 1	1.515

Table S11 (continued)

ERP29	endoplasmic reticulum protein 29	-1.264
FTL	ferritin light chain	-1.214
GCLC	glutamate-cysteine ligase catalytic subunit	-3.214
GSR	glutathione-disulfide reductase	1.57
GSTK1	glutathione S-transferase kappa 1	-1.661
GSTM3	glutathione S-transferase mu 3	Infinity (Up)
GSTO1	glutathione S-transferase omega 1	748.755
HACD3	3-hydroxyacyl-CoA dehydratase 3	1.391
IRS1	insulin receptor substrate 1	-29.26
KRAS	KRAS proto-oncogene, GTPase	10.706
MAP2K1	mitogen-activated protein kinase kinase 1	1.605
MAP2K2	mitogen-activated protein kinase kinase 2	-1.995
MAPK3	mitogen-activated protein kinase 3	-2.007
MAPK14	mitogen-activated protein kinase 14	-1.731
NQO1	NAD(P)H quinone dehydrogenase 1	6.536
NRAS	neuroblastoma RAS viral oncogene homolog	-1.867
PIK3C2B	phosphatidylinositol-4-phosphate 3-kinase catalytic sul	-8.49
PPIB	peptidylprolyl isomerase B	2.116
PRDX1	peroxiredoxin 1	-15.628
PRKCD	protein kinase C delta	-4.107
PRKD3	protein kinase D3	-1.229
SOD1	superoxide dismutase 1, soluble	2.82
SQSTM1	sequestosome 1	1.95
STIP1	stress induced phosphoprotein 1	Infinity (Up)
TXN	thioredoxin	4.359
TXNRD1	thioredoxin reductase 1	-8.208
UBE2K	ubiquitin conjugating enzyme E2 K	-1.306
USP14	ubiquitin specific peptidase 14	-4.101
VCP	valosin containing protein	-3.265

Table S12

IL-8 Signaling

Symbol	Entrez Gene Name	Fold Change
AKT1	AKT serine/threonine kinase 1	1.771
BAX	BCL2 associated X, apoptosis regulator	1.503
CCND1	cyclin D1	-1.822
CCND2	cyclin D2	-2.186
CDH1	cadherin 1	-1.453
CSTB	cystatin B	1.234
CXCL1	C-X-C motif chemokine ligand 1	-1.911
GNA13	G protein subunit alpha 13	-14.665
GNAI3	G protein subunit alpha i3	1.542
GNAS	GNAS complex locus	56
GNB1	G protein subunit beta 1	-4.428
GNB2	G protein subunit beta 2	-4.428
GNB4	G protein subunit beta 4	-4.428
IQGAP1	IQ motif containing GTPase activating protein 1	9.886
IRAK1	interleukin 1 receptor associated kinase 1	-1.669
IRS1	insulin receptor substrate 1	-29.26
ITGAX	integrin subunit alpha X	-1.81
ITGB5	integrin subunit beta 5	-3.977
KRAS	KRAS proto-oncogene, GTPase	10.706
LASP1	LIM and SH3 protein 1	40.465
MAP2K1	mitogen-activated protein kinase kinase 1	1.605
MAP2K2	mitogen-activated protein kinase kinase 2	-1.995
MAP4K4	mitogen-activated protein kinase kinase kinase kinase 4	6.16
MAPK3	mitogen-activated protein kinase 3	-2.007
NRAS	neuroblastoma RAS viral oncogene homolog	-1.867
PAK2	p21 (RAC1) activated kinase 2	2.13
PIK3C2B	phosphatidylinositol-4-phosphate 3-kinase catalytic subunit type 2 beta	-8.49
PLD3	phospholipase D family member 3	1.565

Table S12
(continued)

PRKCD	protein kinase C delta	-4.107
PRKD3	protein kinase D3	-1.229
RAC1	ras-related C3 botulinum toxin substrate 1 (rho family, small GTP binding protein Rac1)	2.185
RACK1	receptor for activated C kinase 1	-5.338
RELA	RELA proto-oncogene, NF-kB subunit	-1.867
RHOA	ras homolog family member A	-2.213
RHOB	ras homolog family member B	1.505
RHOC	ras homolog family member C	-1.371
RHOG	ras homolog family member G	1.386
RHOQ	ras homolog family member Q	-1.253
RHOT2	ras homolog family member T2	-1.304
ROCK1	Rho associated coiled-coil containing protein kinase 1	22.637
ROCK2	Rho associated coiled-coil containing protein kinase 2	8.598
RPS6KB1	ribosomal protein S6 kinase B1	-1.302
VASP	vasodilator-stimulated phosphoprotein	-1.734
VCAM1	vascular cell adhesion molecule 1	-3.316
